# A Non-Redundant Role for *Drosophila* Mkk4 and Hemipterous/Mkk7 in TAK1-Mediated Activation of JNK

**DOI:** 10.1371/journal.pone.0007709

**Published:** 2009-11-03

**Authors:** Peter Geuking, Rajesh Narasimamurthy, Bruno Lemaitre, Konrad Basler, François Leulier

**Affiliations:** 1 Institut für Molekularbiologie, Universität Zürich, Zürich, Switzerland; 2 Centre de Génétique Moléculaire, FRE 3144 CNRS, Centre de Recherche de Gif, Gif-sur-Yvette, France; University of Texas MD Anderson Cancer Center, United States of America

## Abstract

**Background:**

The JNK pathway is a mitogen-activated protein (MAP) kinase pathway involved in the regulation of numerous physiological processes during development and in response to environmental stress. JNK activity is controlled by two MAPK kinases (MAPKK), Mkk4 and Mkk7. Mkk7 plays a prominent role upon Tumor Necrosis Factor (TNF) stimulation. Eiger, the unique TNF-superfamily ligand in *Drosophila*, potently activates JNK signaling through the activation of the MAPKKK Tak1.

**Methodology/Principal Findings:**

In a dominant suppressor screen for new components of the Eiger/JNK-pathway in *Drosophila*, we have identified an allelic series of the *Mkk4* gene. Our genetic and biochemical results demonstrate that Mkk4 is dispensable for normal development and host resistance to systemic bacterial infection but plays a non-redundant role as a MAPKK acting in parallel to Hemipterous/Mkk7 in dTAK1-mediated JNK activation upon Eiger and Imd pathway activation.

**Conclusions/Significance:**

In contrast to mammals, it seems that in *Drosophila* both MAPKKs, Hep/Mkk7 and Mkk4, are required to induce JNK upon TNF or pro-inflammatory stimulation.

## Introduction

The JNK pathway, one of the three major classes of mitogen-activated protein (MAP) kinase pathways (Erk, p38 and JNK), is induced by pro-inflammatory cytokines, such as Tumor Necrosis Factor (TNF) and Interleukin-1 (IL-1), and several forms of environmental stress (e.g. osmotic stress, irradiation, and oxidative stress) [1]. In mammals, JNK is reported to be activated by two MAPK kinases (MAPKK) Mkk4 and Mkk7, with Mkk7 as the major MAPKK in TNF- or IL-1-induced JNK activation while both, Mkk4 and Mkk7, are required for stress induced activation of JNK [2]. In mammals, Mkk7 is a specific activator of JNKs while Mkk4 can also phosphorylate p38 MAPKs [3]. In mice, analysis of the relative contribution of Mkk4 and Mkk7 to JNK activation has been complicated by the fact that *Mkk4* and *Mkk7* single mutants are embryonic lethal [2].

*Drosophila* orthologs of Mkk4 and Mkk7 have been identified [4], [5], [6]. So far, only mutations in *hemipterous/Mkk7* (*hep*), have been isolated [4]. Hep phosphorylates and activates the *Drosophila* JNK, Basket (Bsk) [7] and null mutations in *hep* lead to a defect in dorsal closure, a well characterized process in the *Drosophila* embryo that entirely depends on JNK signaling [4], [7]. In contrast to mammals, *Drosophila* Mkk4 only activates JNK but not p38 in vitro [5], [8], however this remains controversial [6]. To date no mutants for *Drosophila Mkk4* have been identified and its functional relevance towards JNK activation therefore remains elusive. Based on the embryonic lethality of *hep* mutants it is obvious that Mkk4, which is expressed during embryonic development, cannot substitute for Hep function in this process. Although it has been reported that in mammals Mkk4 and Mkk7 may synergistically activate JNK [9], it does not seem to be the case for Hep-mediated Bsk activation during dorsal closure.

In a dominant suppressor screen for new components of the Eiger-JNK-pathway in *Drosophila* [10], we have identified an allelic series of the *Drosophila Mkk4* gene. Our genetic and biochemical experiments now demonstrate a non-redundant role for Mkk4 as a MAPKK acting in parallel of Hep in dTAK1-mediated JNK activation during both Eiger and Imd signaling.

## Results and Discussion

### Mutations in *Mkk4* Suppress Eiger Mediated Small Eye Phenotype

**Figure 1:**
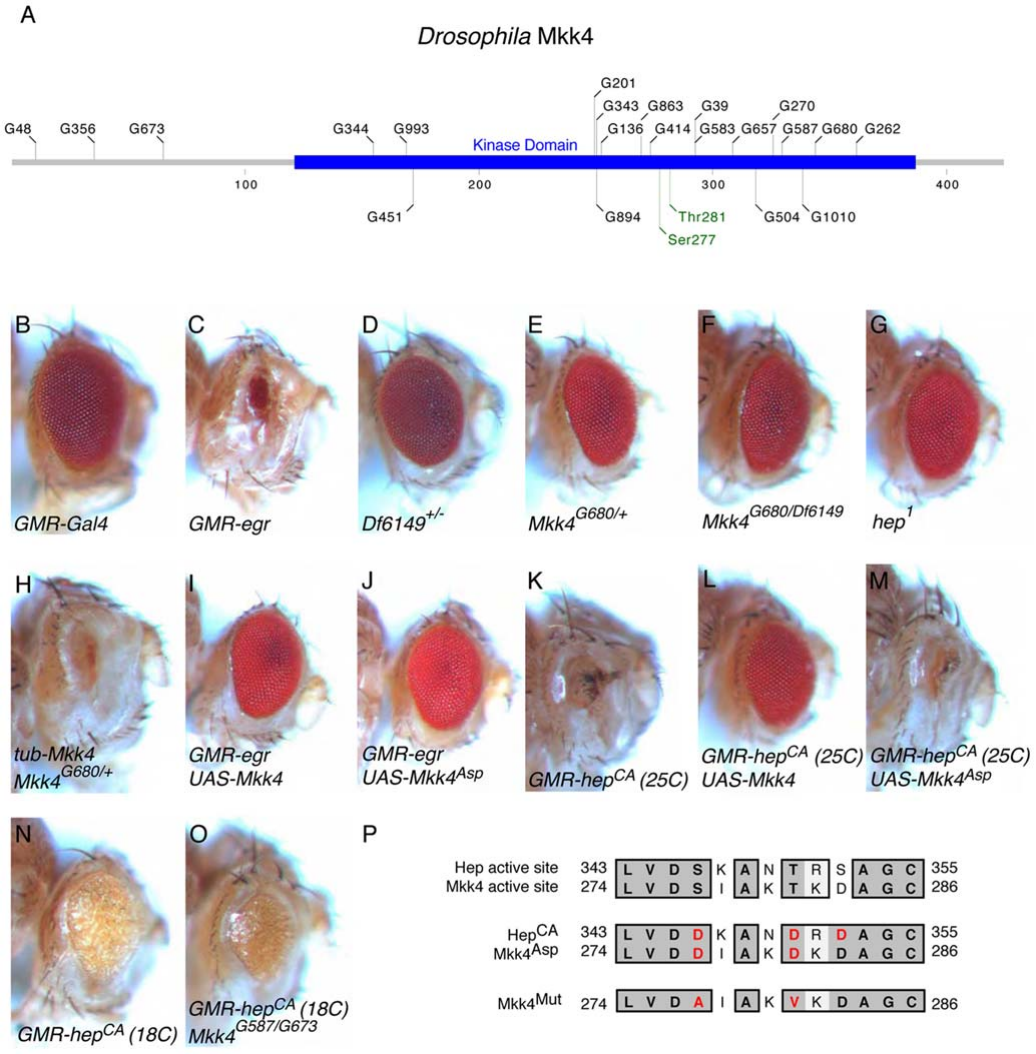
Mkk4 triggers Eiger-mediated small eye phenotype. (A) Schematic representation of Mkk4. Alleles (black) and Ser/Thr phosphorylation sites (green) are indicated. (B)–(J) are in a *GMR-egr* (*GMR-Gal4*,*UAS-egr*) background. (B) *GMR-Gal4/+* control eye. (C) *GMR-egr/+* small eye. (D) *GMR-egr/+*; *Df(3L)Exel6149/+*. (E) *GMR-egr/+*; *Mkk4^G680^/+*. Removing one copy of *Mkk4* suppresses the small eye phenotype. (F) *GMR-egr/+*; *Mkk4^G680^/Df(3L)Exel6149*. Removing both copies of *Mkk4* does not improve the suppression. (G) *hep^1^*; *GMR-egr/+*. Males hemizygous mutant for a hypomorphic *hep* allele display a strong suppression of the small eye. (H) *GMR-egr/tub-Mkk4*; *Mkk4^G680^/+*. A *Mkk4* rescue transgene reverts the dominant suppression observed by loss of one copy of *Mkk4*. (I) *GMR-egr/UAS-Mkk4*. Co-expression of Mkk4 has dominant negative effect on Eiger signal transduction. (J) *GMR-egr/UAS-Mkk4^Asp^*. The same effect is observed for Mkk4^Asp^. (K)–(O) are in a *GMR-hep^CA^* (*GMR-Gal4*,*UAS-hep^CA^*) background. (K) *GMR-hep^CA^/+* (25°C). (L) *GMR-hep^CA^/UAS-Mkk4* (25°C). Co-expression of *Mkk4* also suppresses the small eye phenotype induced by Hep^CA^. (M) *GMR-hep^CA^/UAS-Mkk4^Asp^* (25°C). Co-expression of *Mkk4^Asp^* does not suppress the small eye phenotype induced by Hep^CA^. (N) *GMR-hep^CA^/+* (18°C). Weaker expression of *hep^CA^* leads to a less severe small eye phenotype. (O) *GMR-hep^CA^/+*; *Mkk4^G587^/Mkk4^G673^* (18°C). This phenotype is not suppressed, even when both copies of *Mkk4* are removed. (P) Aligment of the amino-acid sequence of Hep and Mkk4 catalytic region. The mutations introduced in Hep^CA^, Mkk4^Asp^ and Mkk4^Mut^ are indicated in red.

In a dominant suppressor screen for new components of the Eiger-JNK-pathway in *Drosophila* [10], we identified 21 EMS mutations suppressing Eiger-induced cell death in the eye that mapped genetically very close to a deficiency (*Df(3R)Exel6149*) that also suppresses the Eiger-induced small eye phenotype (Figure 1D). This deficiency removes 26 genes including *Mkk4*. We sequenced the coding region of *Mkk4* in those EMS alleles and molecular lesions were detected in all of them. Several mutations create a premature stop codon in the open reading frame and therefore likely represent null alleles (Table 1, Figure 1A). Of note, all the 21 alleles behaved the same and lead to a strong suppression of the Eiger-induced small eye phenotype.

**Table 1:** Mkk4 allelic series.

*Mkk4* allele	Lesion on DNA level (wt – mut)	Lesion on protein level
*G48*	G**C**A – G**T**A	Ala10Lys
*G356*	653bp insertion at Ser38	AA11 of insertion is a STOP
*G673*	**C**AG – **T**AG; T**T**C - T**A**C	Gln66STOP; Phe184Tyr
*G344*	**C**GA - **T**GA	Arg154STOP
*G993*	**G**AT - **A**AT	Asp168Asn
*G451*	**G**TG - **A**TG	Val171Met
*G201*	**G**AT - **A**AT	Asp249Asn
*G343*	**G**TG - **A**TG	Val250Met
*G894*	G**T**G - G**A**G	Val250Asn
*G136*	**C**CG - **T**CG	Pro252Ser
*G863*	G**G**T - G**A**T	Gly269Asp
*G414*	**C**AG - **T**AG	Gln273STOP
*G39*	C**C**G - C**T**G	Pro292Leu
*G583*	**C**CG - **T**CG	Pro292Ser
*G657*	**G**AT - **A**AT	Asp308Asn
*G504*	**G**AG - **A**AG	Glu318Lys
*G270*	C**C**C - C**T**C	Pro325Leu
*G587*	T**G**G - T**A**G	Trp329STOP
*G1010*	5′ splice site intron 3: AG/**G**T - AG/**A**T	-
*G680*	**C**AA – **T**AA	Gln341STOP
*G262*	**G**TG - **A**TG	Val361Met

Molecular lesions identified in *Mkk4*. Alleles are ordered according to their position in the protein.

*Mkk4* mutant flies are viable and do not show obvious morphological defects over *Df(3R)Exel6149* or in heteroallelic combinations. In some cases homozygous lethality is observed which is most likely due to second mutations on the chromosome. The absence of embryonic lethality associated with *Mkk4* loss of function demonstrates that unlike Hep/Mkk7, Mkk4 is not rate limiting for dorsal closure of the *Drosophila* embryo.

Removing a single copy of *Mkk4* leads to a potent suppression of the Eiger-induced small eye phenotype (Figure 1B–E). Removing two copies of *Mkk4* does not significantly enhance this suppression (Figure 1F). Therefore, in this context *Mkk4* mutations are dominant suggesting that *Mkk4* is haplo-insufficient for Eiger-induced small eye phenotype. Introducing a *tubulin-Mkk4* rescue transgene reverts the observed dominant suppression indicating that indeed *Mkk4* is responsible for this effect (Figure 1H). It is important to note that hemizygous males for the hypomorphic *hep^1^* allele also show a very good suppression of the Eiger-induced small eye phenotype [11] (Figure 1G), indicating that in *Drosophila* both MAPKKs, Mkk4 and Hep/Mkk7, are rate limiting for proper transduction of the Eiger signal. This demonstrates that in *Drosophila*, in contrast to mammals, Mkk4 is haplo-insufficient for TNF superfamily ligand (Eiger)-mediated JNK activation.

### Mkk4 Acts as MAPKK for dTAK1 Mediated Activation of JNK

**Figure 2:**
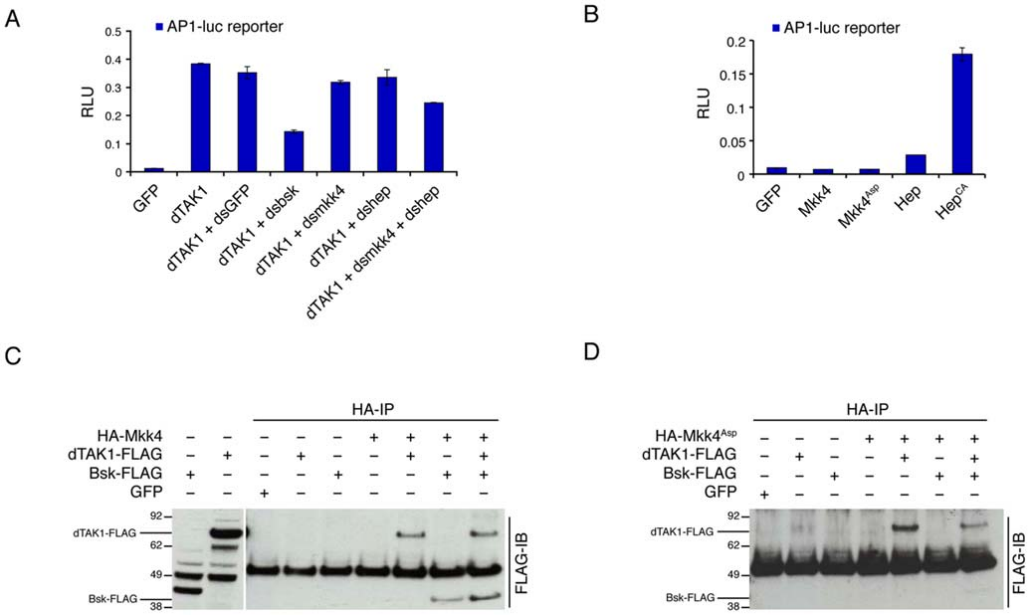
Mkk4 function as a MAPKK between dTAK1 and JNK/Bsk. (A) RNAi against *Mkk4* and *hep* together significantly reduces dTAK1-induced AP-1-luciferase reporter activity. (B) In contrast to Hep and Hep^CA^, Mkk4 and Mkk4^Asp^ do not induce AP-1-luciferase reporter activity on their own. (C) Mkk4 physically interacts with dTAK1 and Bsk. (D) Mkk4^Asp^ still interacts with dTAK1 but no longer binds to Bsk.

**Figure 3:**
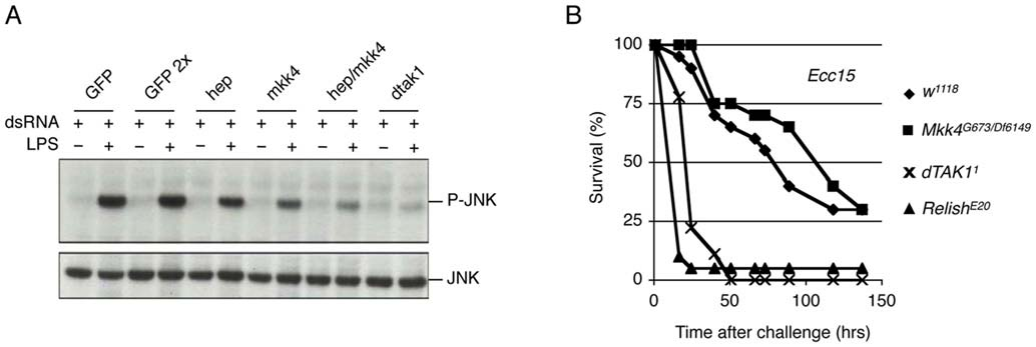
Mkk4 activates the JNK branch of the Imd pathway, not the IKK/Relish branch. (A) RNAi against *Mkk4* and/or *hep* reduces phosphorylated JNK levels induced by commercial LPS in S2 cells. (B) Survival analysis of *w^1118^* (closed diamond), *Mkk4^G673^/Df(3R)Exel6149* (closed square)), *dTAK1^1^* (cross) and *Relish^E20^* (closed triangle) flies upon *Erwinia carotovora carotovora 15* (*Ecc15*) septic injury.

To confirm that Mkk4 indeed acts, like Hep, at the level of a MAPKK in the JNK pathway, we performed epistasis experiments in flies and cells as well as protein interaction studies. Removing one (not shown) or both copies of *Mkk4* does not suppress the small eye phenotype induced by expression of an activated version of *hep* (*hep^CA^*) in the *Drosophila* eye [12] (Figure 1N and O). This result suggests that Mkk4 does not genetically function downstream of Hep. In S2 cells, the expression of the MAPKKK dTAK1 potently activates the JNK pathway, which leads to the activation of the AP1-luciferase-reporter gene (Figure 2A). Co-RNAi against *hep* and *Mkk4* reduces this activity (Figure 2A). However single RNAi treatment against either of the two kinases was not sufficient to reduce the luciferase signal (Figure 2A). In S2 cells the JNK pathway is also activated in a dTAK1 dependent manner upon treatment by commercial preparation of LPS (Figure 3A) [13], [14], [15]. RNAi against either *hep* or *Mkk4* reduces JNK activation upon commercial LPS treatment (Figure 3A) suggesting that both kinases are rate limiting in this situation. In agreement with this, the reduction in phosphorylated JNK levels is enhanced when both kinases are targeted by RNAi at the same time (Figure 3A). This last result confirms previous reports indicating that both, Mkk4 and Hep, are required to mediate JNK activation upon commercial LPS treatment [13], [14]. Taking together our RNAi experiments in S2 cells place Mkk4 downstream of the MAPKKK dTAK1 in the control of JNK, confirming that Mkk4 functions as a classical MAPKK. Further evidence suggesting that Mkk4 indeed acts as a MAPKK was obtained from protein interaction studies. When expressed in S2 cells, N-terminally HA tagged Mkk4 co-immunoprecipitated both, C-terminally FLAG tagged dTAK1 and Bsk (Figure 2C). These results reveal that Mkk4 physically interacts with its upstream kinase dTAK1 as well as with its downstream kinase Bsk.

In contrast to the intrinsic activity of Hep^CA^ (strong) and Hep^WT^ (weak) (Figure 1K,N and 2B) [12], wild type Mkk4 does not activate the JNK pathway when overexpressed in S2 cells (Figure 2B) or in fly eyes (data not shown). Interestingly, wild type Mkk4 has a dominant negative effect when co-expressed with Eiger (Figure 1I) or Hep^CA^ (Figure 1L) in flies. This may stem from its ability to interact with Bsk and dTAK1. Overexpressing Mkk4 may therefore titrate away Bsk and dTAK1. In an attempt to generate a constitutive active Mkk4 (Mkk4^Asp^), we introduced the Ser277→Asp and Thr281→Asp mutations, which corresponds to the mutations that were introduced to generate Hep^CA^ [12] (Figure 1P). Surprisingly, Mkk4^Asp^ is not constitutively active, neither in flies (not shown) nor in S2 cells (Figure 2B). However, expressing Mkk4^Asp^ suppresses *GMR-egr* (Figure 1J) but not *GMR-hep^CA^* (Figure 1M). Finally a kinase dead version of Mkk4 (Mkk4^Mut^) where mutations Ser277→Ala and Thr281→Val were introduced (Figure 1P) behaved identically to Mkk4^WT^ (not shown) suggesting that the kinase activity of Mkk4 is not associated with its dominant negative effect upon overexpression. This effect may rather relate to differential binding ability towards dTAK1 and Bsk of Mkk4^WT^ and Mkk4^Asp^. Indeed in CoIP experiments Mkk4^Asp^ is still able to bind dTAK1 but no longer Bsk (Figure 2D). Altogether our results therefore demonstrate that Mkk4 is a MAPKK acting in parallel of Hep/Mkk7 and downstream of dTAK1 in the activation of Bsk/JNK upon both Eiger expression and Imd pathway activation by commercial LPS.

### Mkk4 Is Dispensable for the Activation of the IKK/Relish Cascade by dTAK1

dTAK1 is an important MAPKKK regulating the activity of both the JNK and IKK/Relish branch of the Imd cascade, a signaling pathway regulating the expression of several immune effectors upon infection [15], [16]. In absence of a functional Imd/IKK/Relish cascade as in dTAK1 mutants, flies are extremely sensitive to systemic infection by Gram-negative bacteria, including *Erwinia carotovora carotovora 15* (*Ecc15*) [16]. Therefore, we investigated if Mkk4 is implicated in the control of the IKK/Relish branch of the Imd pathway. To this end we tested if an *Mkk4* deficiency leads to similar immune phenotypes like *dTAK1* loss of function. We challenged *Mkk4* mutants with *Ecc15* and monitored their survival over time. Figure 3B shows that in contrast to *Relish* and *dTAK1* mutants, *Mkk4* mutants survive like wild-type flies to this challenge. This result therefore suggests that Mkk4 is dispensable for the activation of the Imd/IKK/Relish cascade by dTAK1. Therefore, the involvement of Mkk4 in the Imd cascade is restricted to the dTAK1-mediated activation of the JNK branch (Figure 3A).

### The Egr/dTAK1/Mkk4 Cascade Is Dispensable to Fight Gram-Positive Cocci Infections

**Figure 4:**
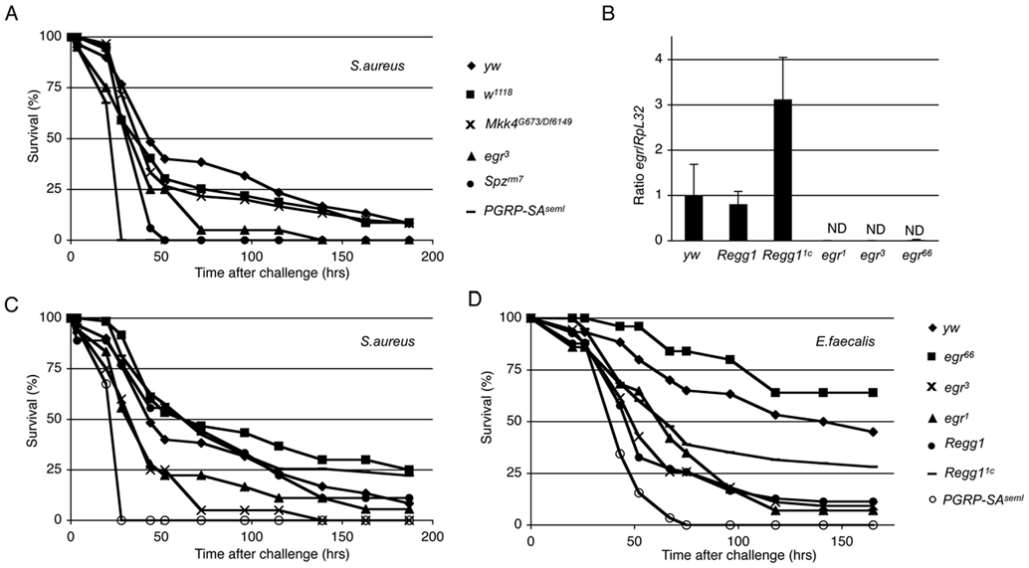
The Egr/dTAK1/Mkk4 cascade is dispensable to fight gram-positive cocci infection. (A) Survival analysis of *yw* (closed diamond), *w^1118^* (closed square), *Mkk4^G673^/Df(3R)Exel6149* (cross), *eiger^3^* (*egr*; closed triangle), *Spz^rm7^* (closed circle) and *PGRP-SA^seml^* (dash) flies upon *Staphylococcus aureus* (*S.aureus*) septic injury. (B) Quantitative RT-qPCR analysis of basal *egr* expression in *yw*, *Regg1*, *Regg1^1C^*, *egr^1^*, *egr^3^* and *egr^66^* adults. ND: Not Detected. *RpL32* was used as the experimental expression standard. Relative DCt*^egr^/*DCt*^RpL32^* ratios of *yw* males were anchored in 1 to indicate fold-induction. Graphs represent the mean and S.D of relative ratios detected in 3 biological repetition of a pool of 15 males. (C–D) Survival analysis of *yw* (closed diamond), *eiger^66^* (closed square), *eiger^3^* (cross), *egr^1^* (closed triangle), *Regg1* (closed circle), *Regg1^1C^* (dash) and *PGRP-SA^seml^* (open circle) flies upon (C) *S.aureus* or (D) *Enterococcus faecalis* (*E.faecalis*) septic injury.

Recently, Schneider and colleagues showed that *eiger* mutants are sensitive to systemic infection by gram-positive cocci, a type of extracellular bacteria [17]. In order to test if this *egr* related process relies on the same signaling cascade as the one activated in the eye upon *egr* expression, we challenged *Mkk4* mutants with *Staphyloccocus aureus*, a gram-positive coccus (Figure 4A). We compared their viability to *egr* mutants and other mutants affecting the Toll signaling pathway, *spz* and *PGRP-SA*, which contribute to the resistance to systemic gram-positive cocci infection [18], [19]. Figure 4A shows that although *egr^3^* mutant show a slight increased susceptibility to this challenge compared to wild-type flies, however this was not as pronounced as *spz* and *PGRP-SA* mutants. Importantly, *Mkk4* mutants behave like wild-type animals in this setting. These results corroborate our data showing that *dTAK1* and *dTAB2* null alleles are not sensitive to Gram-positive cocci infection while being essential to mediate the *egr*-induced small eye phenotype [10], [16] (D. Ferrandon, personal communication; P.G, K.B and F.L unpublished data). Taken together these results therefore suggest that the canonical dTAB2/dTAK1/Mkk4 signaling cassette is not required to mediate the reported function of *egr* in fighting Gram-positive cocci infection.

In contrast to the strong susceptibility reported by Schneider *et al*, our results revealed a mild susceptibility of *egr^3^* alleles to Gram-positive cocci infection. In order to confirm this phenotype, we tested related or independent *egr* alleles (*egr^1^* and *egr^66^*, respectively) as well as the *Regg1* parental line initially used to generate *egr^1^* and *egr^3^* alleles [11], [20]. In addition, we generated a precise excision of the *Regg1* P-element insertion, *Regg1^1c^*, to create an isogenic wild-type control line of the *Regg1*, *egr^1^* and *egr^3^* alleles. As expected, *egr* transcripts were detected by RT-qPCR in all control lines (*yw*, *Regg1* and *Regg1^1c^*) but not in *egr^1^*, *egr^3^* and *egr^66^* mutant lines (Figure 4B). Upon *S.aureus* infection *egr^1^* and *egr^3^* behave similarly and show a mild susceptibility compared to wild-type flies and *PGRP-SA^seml^* mutant flies. However, the *egr^66^* mutants which lack the entire *egr* coding region behave like wild-type controls (Figure 4C). When we repeated this experiment using another, less pathogenic, gram-positive coccus, *Enteroccocus faecalis*, we observed the same pattern of results with *egr^1^*, *egr^3^* and *egr^66^* alleles, with *egr^66^* beeing as susceptible as wild-type flies to *E.faecalis* (Figure 4D). The reduced pathogenicity of *E.faecalis* compared to *S.aureus* revealed that the *Regg1* and *Regg1^1c^* fly lines show a mild susceptibility to this bacterial infection similarly to *egr^1^* and *egr^3^* mutants. Taken together, these results therefore suggest that the observed susceptibility of *egr^1^* and *egr^3^* mutants to Gram-positive cocci is rather due to the genetic background of the *Regg1* line but not associated with *egr* loss of function.

### Conclusion

**Figure 5:**
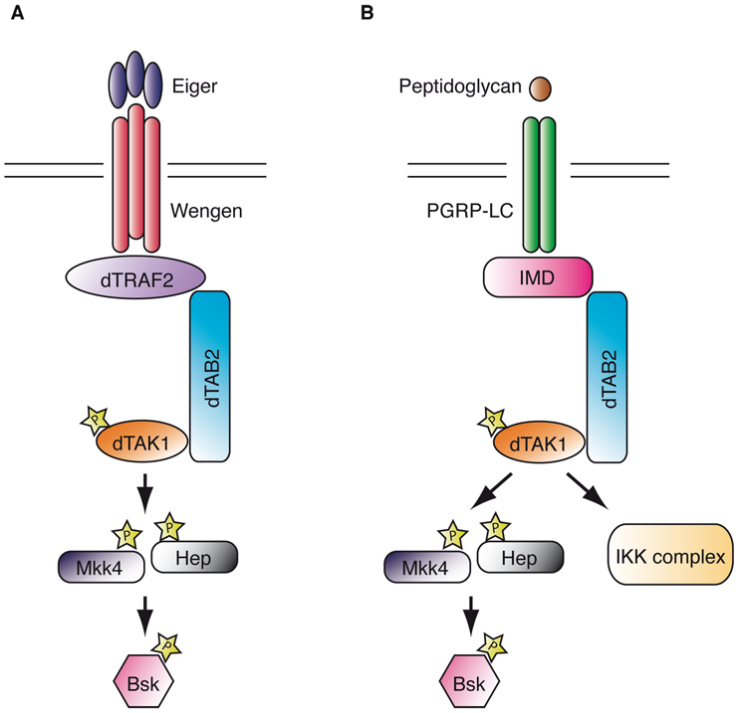
Proposed model for JNK regulation by *Drosophila* Mkk4 and Hemipterous/Mkk7 upon Eiger and Imd pathway activation. (A) Following Eiger binding to Wengen a signaling complex consisting of dTRAF2-dTAB2-dTAK1 is stabilized, which allows dTAK1 activation. Subsequently dTAK1 activates both Mkk4 and Hemipterous/Mkk7, which act non redundantly to activate Bsk/JNK. (B) Following Peptidoglycan recognition PGRP-LC recruits the scaffold protein IMD, which activates by a yet unknown mechanism the dTAB2-dTAK1 complex. dTAK1 subsequently activates directly the IKK complex and both Mkk4 and Hemipterous/Mkk7 which act non redundantly to activate Bsk/JNK.

In this study we have isolated for the first time an allelic series of *Drosophila Mkk4*. Using these mutants we showed that Mkk4 is dispensable for normal development and for host resistance to systemic bacterial infection. Our genetic and biochemical experiments demonstrate a non-redundant role for Mkk4 as a MAPKK acting in parallel to Hep/Mkk7 in dTAK1-mediated JNK activation in the Eiger and Imd pathways (Figure 5). In contrast to mammals, it seems that in *Drosophila* both MAPKKs, Hep/Mkk7 and Mkk4, are required to induce JNK upon TNF or pro-inflammatory stimulation.

## Methods

### Fly Stocks

Fly stocks were maintained on polenta-agar medium at 23°C. *w^1118^* and *yw* fly strains were used as controls when appropriate. Stocks carrying the *UAS-Mkk4* (*Mkk4*) and *tubulin*α*1-Mkk4* transgene were obtained by standard *P*-element-induced transformation. *dTAK1^1^*, *Relish^E20^*, *spz^rm7^*, *PGRP-SA^seml^*, *Regg1*, *egr^1^*, *egr^3^*, *egr^66^* and *hep^1^* fly strains were described previously [4], [11], [20], [21]. *Regg1^1c^* was generated by classical transposase-mediated precise excision of the *Regg1* P-element. *Df(3R)Exel6149* was obtained from Bloomington stock center,. UAS-*hep^CA^*, GMR-*gal4* and UAS-*egr* were previously described [10], [12].

### Genetic Mapping of the Alleles

From our dominant suppressor screen no lethal complementation group on the third chromosome could be identified (10). Based on this observation we decided to combine two strategies in order to map dominant suppressor mutations on the third chromosome. First, we screened the whole Exelixis deficiency kit [22] for dominant suppressors of the Egr-induced small eye phenotype. *Df(3R)Exel6149* was selected as a dominant suppressor deficiency. This deficiency removes 26 genes including *Mkk4* and maps to the cytological location 85A [22]. Second, we performed a classic genetic mapping by meiotic recombination. In absence of a homozygous phenotype we made use of the dominant suppressor phenotype in our sensitized background. To this end, three RFP-(red fluorescent protein)-marked insertions at positions 62B, 85E and 92A were used as genetic markers (one of them located close to *Df(3R)Exel6149* at position 85A, which was isolated as a dominant suppressor from the deficiency screen). Chromosomes carrying a suppressor mutation were allowed to recombine with the RFP-marked chromosome in females. These virgins were crossed back to GMR-egr/CyO males. The number of non-CyO RFP(+) progeny (sorted under a fluorescent binocular) with a suppressed eye phenotype in relation to the number of non-CyO RFP(+) progeny with a small eye reflects the relative genetic distance to the RFP insertion. In stocks carrying a suppressor mutation that mapped very close to the RFP(+) insertion at 85E (only 1-3% recombination frequency between suppressor mutation and RFP(+) at 85E), and therefore also close to *Df(3R)Exel6149* at 85A, the *Mkk4* gene was checked for point mutations by sequencing. The *Mkk4* gene was chosen because it was the most evident candidate from the 26 genes deleted in *Df(3R)Exel6149*.

### Transgenes

The *Mkk4* full-length cDNA (RE70055) was cloned into pUAST [23] and into a vector containing the *tubulin*α*1* promoter [24], respectively. For *UAS-Mkk4^Asp^* and *UAS-Mkk4^mut^* the corresponding mutations were introduced by classical site directed mutagenesis.

### Sequencing

Genomic DNA was amplified by PCR using evenly spaced primers in the *Mkk4* coding region. PCR products were analyzed by standard sequencing.

### *Drosophila* Cell Culture and Transfection

Schneider (S2) cells were cultured in Schneider's Drosophila medium (Invitrogen, San Diego) supplemented with 10% fetal calf serum and 1% penicillin/streptomysin at 25°. Cells were transfected with expression vectors, using Cellfectin (Invitrogen) according to the manufacturer's protocol. Expression vectors: pUAST-*Mkk4*, pUAST-HA-*Mkk4*, pUAST-*Mkk4^Asp^*, pUAST-HA-*Mkk4^Asp^*, pUAST-*dTAK1*, pUAST-*dTAK1*-FLAG, pUAST-*hep*, pUAST-*hep^CA^*, pUAST-*bsk*-FLAG, ptub-Gal4, pUAST-GFP.

### Immunoprecipitation and Immunoblotting

S2 cells (0.75×10^6^ cells/well) were seeded into a 12-well plate. One day after seeding cells were transfected with the indicated expression vectors. Forty-eight hours after transfection the cells were harvested and lysed in lysis buffer containing 150 mm NaCl, 50 mm Tris-HCl (pH 8.0), 1% Nonidet P-40, 0.5% deoxycholic acid, and protease inhibitors (Complete Mini; Roche, Indianapolis). Lysates were mixed with an anti-HA antibody and 25 µl of Protein-A sepharose beads and allowed to rotate at 4°C overnight. The beads were then collected and washed with the lysis buffer four times. Proteins were eluted from the beads and resolved on a 4–12% NUPAGE gel system (Invitrogen) and transferred to a nitrocellulose membrane. After blocking, the membrane was incubated with anti-FLAG M2 antibody (Sigma) followed by appropriate secondary antibodies conjugated with horseradish peroxidase (HRP). Signals were detected with ECL reagents (Amersham, Arlington Heights, IL).

### LPS Treatment

S2 cells were treated with dsRNA (15 µg/10^6^ cells) and split into two halfs. One half was left untreated and the other half was treated with lipopolysaccharide (LPS) (Sigma) at a concentration of 50 µg/ml for 10 min (note that commercial preparation of LPS contains peptidoglycan, which potently induces the Imd signaling cascade [21]). The cells were then lysed in lysis buffer. The lysates were analyzed by immunoblotting to detect phosphorylated JNK (anti-P-JNK, Promega, Madison, WI) and JNK (anti-JNK, Santa Cruz Biotechnologies, Santa Cruz, CA).

### Luciferase Assay

S2 cells (0.4×10^6^ cells/well) were seeded into a 24-well plate. One day after seeding cells were transfected with an AP1-luciferase reporter plasmid along with the indicated expression vector. The total DNA concentration (1 µg) was kept constant by supplementing with empty vector. Forty-eight hours after transfection, cells were harvested, lysed in passive lysis buffer, and luciferase activity was measured using the dual luciferase assay system (Promega). The values shown reflect the relative luciferase activity: the ratio of firefly (AP1 luciferase) and *tub-renilla* luciferase activity of one representative experiment in which each transfection was made in duplicate.

### Double-Stranded RNA Production

Double-stranded RNA (dsRNA) was prepared as described by the Dixon lab [25]. Briefly, using PCR products as templates, the MEGASCRIPT T7 transcription kit (Ambion, Austin, TX) was used to produce RNA according to the manufacturer's protocol. RNA products were ethanol precipitated and resuspended in DEPC-treated water. dsRNA was generated by annealing at 65° for 30 min followed by slow cooling to room temperature. The following sets of forward and reverse primers were used (T7 sequences are not indicated):

*Mkk4*: sense 5′-caatcccccggatcagctaag-3′; antisense 5′-cacatcccgatggataatctttagc-3′

*hep*: sense 5′-gcaagtacattgtcaagtgcc-3′; antisense 5′-tggagcgttggatcgccattgg-3′

*Bsk*: sense 5′-cgccgcaaaggaacttgg-3′; antisense 5′-tcagcatcataccacacg-3′

*dTAK1*: sense 5′-gatgaccaacaatcgcgg-3′; antisense 5′-ggcgctgagtggcctcagc-3′

*GFP*: sense 5′-gaacttttcactggagttgtcc-3′; antisense 5′-gccatgtgtaatcccagcagc-3′

### Quantitative Real-Time PCR

SYBR Green quantitative real-time PCR analysis was performed as previously described [21]. Primer pairs for *egr* (sense: 5′-TAATCTCCAGCAGCGT-3′, and antisense 5′-GTAGTCTGCGCCAACA-3′) and *RpL32* (sense, 5-GAC GCT TCA AGG GAC AGT ATC TG-3, and antisense, 5′-AAA CGC GGT TCT GCA TGA G-3′) were used to detect target gene transcripts. The amount of *egr* mRNA detected was normalized to control *RpL32* mRNA values. Normalized data was used to quantify the relative levels of a given mRNA according to cycling threshold analysis (ΔCt). Relative ΔCt*^egr^/*ΔCt*^RpL32^* ratios of WT controls were anchored in 1 to indicate fold-induction. Graphs represent the mean and S.D of relative ratios detected in 3 biological repetition of a pool of 15 males.

### Bacterial Strains and Infection Experiments

Systemic infections were performed by pricking 60 adult males of 4 to 7 days old in the lateral thoracic region with a thin needle previously dipped into a concentrated pellet of the following bacteria. *Erwinia carotovora carotovora* 15 (Optical Density at 600 nm (OD) 170, 29°C); *Enterococcus faecalis* (OD 10, 25°C) or *Staphylococcus aureus* (OD 10, 25°C). Flies were incubated at the indicated temperatures and their survival was monitored twice every day.
